# Evaluation of cannabinoid type 2 receptor expression and pyridine-based radiotracers in brains from a mouse model of Alzheimer’s disease

**DOI:** 10.3389/fnagi.2022.1018610

**Published:** 2022-09-30

**Authors:** Vasil Kecheliev, Francesco Spinelli, Adrienne Herde, Achi Haider, Linjing Mu, Jan Klohs, Simon M. Ametamey, Ruiqing Ni

**Affiliations:** ^1^Institute for Regenerative Medicine, University of Zurich, Zurich, Switzerland; ^2^Department of Chemistry and Applied Biosciences, ETH Zurich, Zurich, Switzerland; ^3^Department of Nuclear Medicine, University Hospital Zurich, Zurich, Switzerland; ^4^Institute for Biomedical Engineering, University of Zurich and ETH Zurich, Zurich, Switzerland

**Keywords:** Alzheimer’s disease, positron emission tomography, astrocyte, microglia, cannabinoid type 2 receptor (CB_2_R), neuroinflammation, autoradiography

## Abstract

Neuroinflammation plays an important role in the pathophysiology of Alzheimer’s disease. The cannabinoid type 2 receptor (CB_2_R) is an emerging target for neuroinflammation and therapeutics of Alzheimer’s disease. Here, we aim to assess the alterations in brain CB_2_R levels and evaluate novel CB_2_R imaging tracers in the arcAß mouse model of Alzheimer’s disease amyloidosis. Immunohistochemical staining for amyloid-ß deposits (6E10), microgliosis (anti-Iba1 and anti-CD68 antibodies), astrocytes (GFAP) and the anti-CB_2_R antibody was performed on brain slices from 17-month-old arcAß mice. Autoradiography using the CB_2_R imaging probes [^18^F]RoSMA-18-d6, [^11^C]RSR-056, and [^11^C]RS-028 and mRNA analysis were performed in brain tissue from arcAß and non-transgenic littermate (NTL) mice at 6, 17, and 24 months of age. Specific increased CB_2_R immunofluorescence intensities on the increased number of GFAP-positive astrocytes and Iba1-positive microglia were detected in the hippocampus and cortex of 17-month-old arcAß mice compared to NTL mice. CB_2_R immunofluorescence was higher in glial cells inside 6E10-positive amyloid-ß deposits than peri-plaque glial cells, which showed low background immunofluorescence in the hippocampus and cortex of 17-month-old arcAß mice. *Ex vivo* autoradiography showed that the specific binding of [^18^F]RoSMA-18-d6 and [^11^C]RSR-056 was comparable in arcAß and NTL mice at 6, 17, and 24 months of age. The level of *Cnr2* mRNA expression in the brain was not significantly different between arcAß and NTL mice at 6, 17, or 24 months of age. In conclusion, we demonstrated pronounced specific increases in microglial and astroglial CB_2_R expression levels in a mouse model of AD-related cerebral amyloidosis, emphasizing CB_2_R as a suitable target for imaging neuroinflammation.

## Introduction

Abnormal accumulation of amyloid-beta (Aβ) aggregates in Alzheimer’s disease (AD) leads to a cascade of pathophysiological changes, including neuroinflammation, microvascular alterations, synaptic dysfunction, and neuronal loss. Neuroinflammation, including gliosis and increased levels of complements, cytokines, and chemokines, plays an important role in the development of AD (Rapic et al., 2013; Heneka et al., 2015; López et al., 2018; Xin et al., 2020). Microglia are resident macrophages in the central nervous system (CNS) that are important for maintaining brain homeostasis (Heneka et al., 2015) but have also been implicated in the pathophysiology of AD (Deczkowska et al., 2018; Kiani Shabestari et al., 2022). Increased numbers of astrocytes and microglia were observed in the vicinity of Aβ plaques in post-mortem AD mouse model brains and patients with AD (Heneka et al., 2015). Recent single-cell sequencing transcriptomics for disease-associated microglia (DAM) indicates the presence of transcriptionally distinct and neurodegeneration-specific microglial profiles with potential significance in AD signatures, including TREM2, CD33, and ApoE (Keren-Shaul et al., 2017; Deczkowska et al., 2018; Song and Colonna, 2018; Grubman et al., 2021).

Positron emission tomography (PET) ligands for detecting neuroinflammation microgliosis and astrocytosis in AD for understanding the disease mechanism. Early neuroinflammation has been reported in AD and in amyloidosis animal models (Rodriguez-Vieitez et al., 2015; Kreisl et al., 2020; Biechele et al., 2021; Ni et al., 2021b). Among these, PET ligands for translocator protein (TSPO) are the most widely used for detecting neuroinflammation. Previous TSPO PET imaging studies have shown microglial activation preceding Aβ deposition in several animal models, such as APP23, J20, APPSL70, App*^NL–G–F^*, and PS2APP mice (Sacher et al., 2019; Biechele et al., 2021). However, limitations in the complex cellular locations, polymorphisms, and non-specific binding of TSPO and whether TSPO measures microglial proliferation or activation remain to be addressed (Leng and Edison, 2021; Zhou et al., 2021). Novel specific PET tracers for visualizing microgliosis, especially the DAM subtype, are highly desired.

Cannabinoid type 2 receptors (CB_2_Rs) are mainly expressed by immune cells, including monocytes and macrophages. In the CNS, CB_2_Rs are mainly expressed on microglia at low levels under physiological conditions and are upregulated in acute inflammatory conditions, such as ischemic stroke (Cristino et al., 2020). CB_2_Rs are essential to induce Toll-like receptor-mediated microglial activation (Reusch et al., 2022). Activation of CB_2_R offers neuroprotective effects, such as reducing Aβ-induced neuronal toxicity (Köfalvi et al., 2016; Navarro et al., 2018; Wang et al., 2018b; Scheiner et al., 2019; Zhao et al., 2020), suppressing microglial activation (Ehrhart et al., 2005; Ramírez et al., 2005), restoring cognitive capacity (Wu et al., 2017), and ameliorating novel object recognition in animal models of amyloidosis (Li et al., 2019). Thus, CB_2_R has been of therapeutic interest in AD (Köfalvi et al., 2016). However, the expression levels of CB_2_R in animal models of AD amyloidosis have not been extensively characterized. CB_2_R has been shown to be increased and involved in Aβ pathology in 5 × FAD (López et al., 2018; Zhang and Chen, 2018) and J20 mouse models of AD amyloidosis (Koppel et al., 2014) but reduced in the brains of 3 × Tg mice (with both Aβ and tau pathology) and in aging C57B6 mice (Wang et al., 2018a).

Several CB_2_R ligands have been developed and evaluated (Ni et al., 2019b), including [^11^C]NE40 (Vandeputte et al., 2012), [^11^C]A-836339 (MDTC) (Pottier et al., 2017; Du et al., 2022), [^18^F]MA3 (Attili et al., 2019), [^18^F]FC0324 (Caillé et al., 2017), [^18^F]JHU94620 (Moldovan et al., 2016), [^18^F]LU13 (Gündel et al., 2022), [^18^F]DM102 (Modemann et al., 2022), [^18^F]CRA13 (Hassan et al., 2020), [^11^C]RS-016 (Meletta et al., 2017), [^11^C]RS-028 (Haider et al., 2018), [^11^C]RSR-056 (Slavik et al., 2015), and [^18^F]RoSMA-18-d6 (Haider et al., 2020). Thus far, only one in-human *in vivo* CB_2_R PET using [^11^C]NE40 (Ahmad et al., 2016) in patients with AD and healthy controls has been reported, showing no group difference. Only the tracer [^11^C]A-836339 has been evaluated in an AD animal model: Increased [^11^C]A-836339 uptake was observed in the cortex, cerebellum and whole brain of J20 mice compared to wild-type mice (Savonenko et al., 2015); another [^11^C]A-836339 microPET study showed that the uptake was blockable in the cortex of APP/PS1 mice (Horti et al., 2010).

The aim of the current study was to assess the alterations in CB_2_R and distribution in the brain of the arcAß mouse model of AD amyloidosis and to evaluate the recently developed pyridine-derived CB_2_R tracers [^11^C]RS-028, [^18^F]RoSMA-18-d6, and [^11^C]RSR-056, which exhibit subnanomolar affinity and high selectivity toward CB_2_R (Ni et al., 2021a).

## Materials and methods

### Animals

Twenty transgenic arcAβ mice overexpressing the human APP695 transgene containing the Swedish (K670N/M671L) and Arctic (E693G) mutations under control of the prion protein promoter at 6, 17, and 24 months of age and 20 age-matched non-transgenic littermates (NTLs) of both sexes were used in this study (Knobloch et al., 2007; Merlini et al., 2011; Ni et al., 2022). The arcAβ mouse model exhibits parenchymal plaque as well as cerebral amyloid angiopathy and shows impaired cerebrovascular functions (Ni et al., 2018, 2019a). Paper tissue and red mouse house (Tecniplast^®^, Buguggiate VA, Italy) shelters were placed in cages for environmental enrichment. All experiments were performed in accordance with the Swiss Federal Act on Animal Protection and were approved by the Cantonal Veterinary Office Zurich ZH082/18.

For mRNA and autoradiography, arcAβ and age-matched NTL mice at 6, 17, and 24 months of age were anesthetized under 5% isoflurane and decapitated. One brain hemisphere from arcAß mice and NTLs was immediately frozen in liquid nitrogen and stored at −80°C as described earlier (Ni et al., 2021a). The other half of the brain hemisphere was embedded in Tissue Tekv (Sakura^®^ Finetek, Torrance, CA, USA), frozen, and stored at −80°C for autoradiography. For immunofluorescence staining, mice were perfused under ketamine/xylazine/acepromazine maleate anesthesia (75/10/2 mg/kg body weight, i.p. bolus injection) with ice-cold 0.1 M phosphate-buffered saline (PBS, pH 7.4, Sigma Aldrich, Burlington, VT, USA) and 4% paraformaldehyde in 0.1 M PBS (pH 7.4), fixed for 2 h in 4% paraformaldehyde (pH 7.4), and then stored in 0.1 M PBS (pH 7.4) at 4°C.

### mRNA isolation and real-time polymerase chain reaction

Total mRNA isolation of the brain tissue from arcAβ and age-matched NTL mice at 6, 17, and 24 months of age was performed according to the protocols of the Isol-RNA Lysis Reagent (5 Prime Sciences, Montreal, Canada) and the bead-milling TissueLyser system (Qiagen, Hilden, Germany) (Ni et al., 2021a). A QuantiTect^®^ Reverse Transcription Kit (Qiagen) was used to generate cDNA. The primers (Microsynth, Balgach, Switzerland) used for quantitative polymerase chain reaction (qPCR) are summarized in Supplementary Table 1. Quantitation of *Cnr2* mRNA expression was performed with the DyNAmo Flash SYBR^®^ Green qPCR Kit (Thermo Fisher Scientific, Waltham, MA, USA) using a 7900 HT Fast Real-Time PCR System (Applied Biosystems, Waltham, MA, USA). The amplification signals were detected in real time, which permitted accurate quantification of the amounts of the initial RNA template over 40 cycles according to the manufacturer’s protocol. All reactions were performed in duplicate within three independent runs, and each reaction was normalized against the expression of beta-actin. Quantitative analysis was performed using SDS Software (v2.4) and a previously described 2^–ΔΔCt^ quantification method (Livak and Schmittgen, 2001). The specificity of the PCR products of each run was determined and verified with SDS dissociation curve analysis.

### Immunofluorescence

For immunohistochemical analysis, brain tissue from arcAβ and age-matched NTL mice at 17 months of age was used. Coronal brain sections (40 μm) were cut around Bregma 0 to −2 mm and stained with anti-Aβ antibody 6E10, anti-ionized calcium-binding adapter 1 (Iba1) and anti-CD68 for microgliosis, GFAP for astrocytes and anti-CB_2_R antibody as previously described (Kecheliev et al., 2022; Supplementary Table 2). The CB_2_R antibody used in this study targets amino acid region 300–350 on murine CB_2_R protein expressed by cells of hematopoietic origin. Sections were mounted with Prolong Diamond mounting media. Imaging occurred at ×20 magnification using an Axio Observer Z1 slide scanner (Zeiss, Oberkochen, Germany) using the same acquisition settings for all brain slices and at ×63 magnification using a Leica SP8 confocal microscope (Leica, Wetzlar, Germany). The images were analyzed by a person blinded to the genotype using QuPath and ImageJ (NIH, USA). The colocalization of CB_2_R with plaque (6E10 channel), GFAP+ astrocytes or Iba1+ microglia in the cortex and hippocampus was determined on ×63-magnification images. The amount of CB_2_R immunofluorescence within these masks was determined by measuring the mean CB_2_R intensity as well as its integrated density (factor of area and average intensity).

### Radiosynthesis and autoradiography

[^18^F]RoSMA-18-d6 (affinity Ki = 0.8 nM, CB_2_R/CB_1_R > 12,000), [^11^C]RSR-056 and [^11^C]RS-028 were synthesized and purified as described previously (Slavik et al., 2015; Haider et al., 2018, 2020; Ni et al., 2021a) and formulated with 5% ethanol in water. The molar activities were 156–194, 52.3, and 86.7–178 GBq/μmol for [^18^F]RoSMA-18-d6, [^11^C]RSR-056, and [^11^C]RS-028, respectively. The radiochemical purity for all three radioligands was >99%. Autoradiography was performed as described previously (Haider et al., 2020). Dissected mouse brains were embedded in Tissue Tek, cut into 10 μm thick sagittal sections on a cryostat (Cryo-Star HM-560MV; Microm, Thermo Scientific, Waltham, MA, USA) and stored at −80°C. We first compared the percentage of specific binding of [^18^F]RoSMA-18-d6, [^11^C]RSR-056, and [^11^C]RS-028 using brain tissue slices from the same arcAβ mice at 17 months of age (*n* = 3). Next, brain tissue slices from the arcAβ and age-matched NTL mice at 6 months of age (*n* = 5, 6), 17 months of age (*n* = 4, 5), and 24 months of age (*n* = 5, 5) were used in [^18^F]RoSMA-18-d6 autoradiography. Brain tissue slices from the arcAβ and age-matched NTL mice at 6 months of age (*n* = 3, 3), 17 months of age (*n* = 3, 3), and 24 months of age (*n* = 3, 3) were used in [^11^C]RSR-056 autoradiography. Autoradiography binding data from the same mice was analyzed using correlation analysis.

For [^18^F]RoSMA-18-d6 and [^11^C]RSR-056 autoradiography, slices were thawed on ice and preconditioned in ice-cold buffer (pH 7.4) containing 50 mM TRIS Sigma Aldrich, Burlington, VT, USA, 5 mM MgCl_2_ Sigma Aldrich, Burlington, VT, USA, and 0.1% fatty acid-free bovine serum albumin (BSA, Sigma Aldrich, Burlington, VT, USA). The tissue slices were dried and then incubated with 1 ml of the corresponding radioligand (0.5 nM [^18^F]RoSMA-18-d6 and 2 nM [^11^C]RSR-056) for 15 min at room temperature in a humidified chamber. For blockade conditions, the selective CB_2_R antagonist GW405833 (10 μM, Sigma Aldrich, Burlington, VT, USA) was added to the solution containing the radioligand. A standard was place to calibrate the radioligand concentration for calculation of protein level. The slices were washed with ice-cold washing buffer (pH 7.4) containing 50 mM TRIS, 5 mM MgCl_2_, 0.1% fatty acid-free BSA, and ice-cold distilled water. For [^11^C]RS-028 autoradiography, slices were thawed on ice and preconditioned in ice-cold buffer (pH 7.4) containing 50 mM TRIS, 5 mM MgCl_2_, 2.5 mM ethylenediaminetetraacetic acid (EDTA, Sigma Aldrich, Burlington, VT, USA) and 0.1% fatty acid-free BSA. The tissue slices were dried and then incubated with 1 ml of the [^11^C]RS-028 (3 nM) for 15 min at room temperature in a humidified chamber. For blockade conditions, the selective CB_2_R antagonist GW405833 (10 μM) was added to the solution containing the radioligand. A standard was place to calibrate the radioligand concentration for calculation of protein level. Specific binding was calculated as the difference between total and blockade condition. After drying, the slices were exposed to a phosphorimager plate (FUJIFILM, Tokyo, Japan) for 30 min, and the film was scanned in a BAS5000 reader (FUJIFILM).

### Statistics

Group comparisons in multiple brain regions were performed by using two-way ANOVA with Sidak’s *post-hoc* analysis (GraphPad Prism 9, GraphPad, San Diego, CA, USA). Comparisons for CB_2_R inside plaque, peri-plaque and parenchymal were performed by using one-way ANOVA with Tukey’s *post-hoc* analysis. All data are presented as the mean ± standard deviation. Significance was set at *p < 0.05.

## Results

### Increased cannabinoid type 2 receptor expression with proliferation of microglia and astrocytes in the brains of arcAβ compared to non-transgenic littermate

**FIGURE 1:**
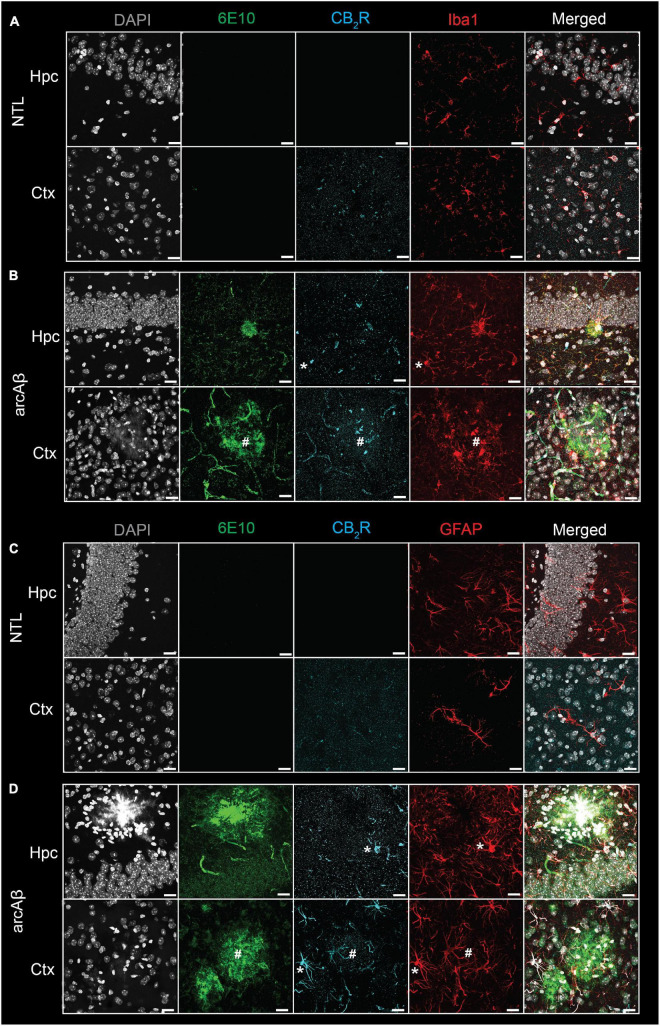
Increased cannabinoid type 2 receptor (CB_2_R) in microglia and astrocytes associated with amyloid-beta deposits in 17-month-old arcAβ mice. **(A,B)** Brain tissue sections of non-transgenic littermate (NTL, *n* = 3) and arcAβ mice (*n* = 3) were stained for Aβ (6E10 antibody, green), CB_2_R (cyan), and Iba1 (red) in the hippocampus (Hpc) and cortex (Ctx). Increased CB_2_R and Iba1 immunoreactivity inside and surrounding the plaque. **(C,D)** Staining for Aβ (6E10, green), CB_2_R (cyan), and GFAP (red) in the Hpc and Ctx. Nuclei were counterstained with DAPI (white). Increased CB_2_R and GFAP immunoreactivity inside and surrounding Aβ plaques. *Localization of CB_2_R on microglia or astrocytes outside plaque, ^#^Colocalization of CB_2_R on microglia or astrocytes within plaque. Scale bar = 20 μm. CB_2_R immunoreactivity was detected on both microglia and astrocytes.

**FIGURE 2:**
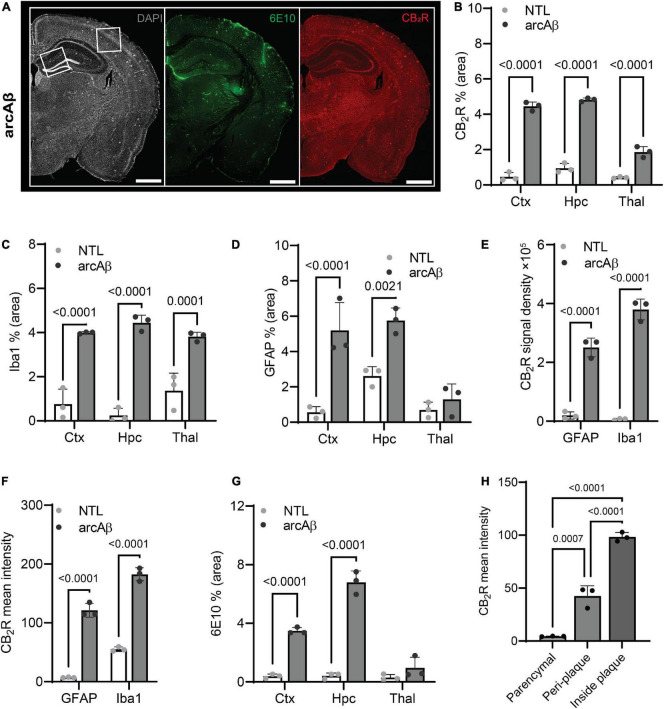
Quantification of microgliosis-, astrocytosis-, cannabinoid type 2 receptor (CB_2_R), and Aβ plaque-associated enrichment in 17-month-old arcAβ mice. **(A)** Representative CB_2_R (red) and 6E10 (Aβ, green) staining in half hemisphere of one arcAβ mouse brain. White boxes in panel **(A)** indicate the regions including cortex (Ctx), cornu ammonus 1 (CA1) region of the hippocampus (Hpc) that zoomed-in in Figure 1. Scale bar = 1 mm. **(B)** Increased CB_2_R (% area) in the Ctx, Hpc, and thalamus (Thal) of arcAβ mice (*n* = 3) compared to non-transgenic littermates (NTLs, *n* = 3). **(C,D)** Increased levels of Iba1 (% area) in the Ctx, Hpc, and Thal and GFAP (% area) in the Ctx and Hpc of arcAβ mice (*n* = 3) compared to NTL (*n* = 3). **(E,F)** Increased CB_2_R signal density and mean signal intensity on both GFAP+ astrocytes and Iba1+ microglia of arcAβ mice (*n* = 3) compared to NTL (*n* = 3). **(G)** Increased 6E10 staining of Aβ plaque in the Ctx and Hpc of arcAβ mice (*n* = 3) compared to NTL (*n* = 3). **(H)** CB_2_R mean signal intensity on the glia inside plaque is higher than peri-plaque, with low background signal in the parenchymal of arcAβ mice. Data are presented as the mean ± standard deviation.

First, the regional CB_2_R level, the cellular source and the expression of CB_2_R (mean immunofluorescence on the area occupied by the selected marker) were assessed in the brains of arcAβ mice and NTL mice at 17 months of age. CB_2_R immunofluorescence intensity was increased approximately 4–10-fold in the cortex (4.45 ± 0.25 vs. 0.46 ± 0.25, *p* < 0.0001), hippocampus (4.82 ± 0.10 vs. 0.95 ± 0.27, *p* < 0.0001), and thalamus (1.87 ± 0.31 vs. 0.41 ± 0.05, *p* < 0.0001) of arcAß mice compared to NTL mice at 17 months of age (*n* = 3 per group) (Figures 1, 2A,B). The background signal of CB_2_R is low in the parenchyma (outside astrocytes/microglia) (Figures 1, 2H). CB_2_R signal density was calculated as the factor of average intensity and area of fluorescent image pixels.

Colocalization analysis indicated that CB_2_R signal density was upregulated on both Iba1^+^ microglia (379855.49 ± 35254.48 vs. 6486.02 ± 2773.22, *p* < 0.0001) and GFAP^+^ astrocytes (250994.60 ± 31974.33 vs. 19568.63 ± 12282.96, *p* < 0.0001) in the brains of arcAß mice compared to NTL mice at 17 months of age (*n* = 3 per group) (Figure 2E). Furthermore, the average CB_2_R signal intensity is increased in image pixels indicating both Iba1^+^ microglia (55.25 ± 3.76 vs. 182.44 ± 11.10, *p* < 0.0001) and GFAP^+^ astrocytes (121.37 ± 11.80 vs. 7.02 ± 0.79, *p* < 0.0001) of arcAß mice compared to NTL mice at 17 months of age (*n* = 3 per group) (Figure 2F).

### Increased cannabinoid type 2 receptor associated with 6E10-positive Aβ plaque in the brains of arcAβ compared to non-transgenic littermate

Increased 6E10 immunofluorescence intensity was observed in the cortex (3.48 ± 0.22 vs. 0.39 ± 0.15, *p* < 0.0001) and hippocampus (6.80 ± 0.77 vs. 0.42 ± 0.20, *p* < 0.0001) of arcAß mice compared to NTL mice at 17 months of age (*n* = 3 per group) and was comparable in the thalamus (0.95 ± 0.73 vs. 0.30 ± 0.20, *p* = 0.2936) (Figures 1, 2G). In the brains of arcAß mice at 17 months of age, CB_2_R immunofluorescence was located on microglia and astrocytes both inside/within plaques (Figure 1). The intra-plaque (98.27 ± 4.31 *p* < 0.0001) and peri-plaque (42.38 ± 9.84, *p* = 0.0007) glial CB_2_R levels were both 20-fold and 10-fold that in the parenchyma (4.5 ± 0.24) of the arcAβ mice (*n* = 3 per group), respectively. The glial-CB_2_R mean fluorescence intensity inside plaque was higher than that located peri-plaque of the arcAβ mice (*p* < 0.0001, *n* = 3 per group) (Figures 1, 2H).

### Increased Iba1+ and CD68+ microglia and GFAP+ astrocytes in the brains of arcAβ mice compared to non-transgenic littermate mice

**FIGURE 3:**
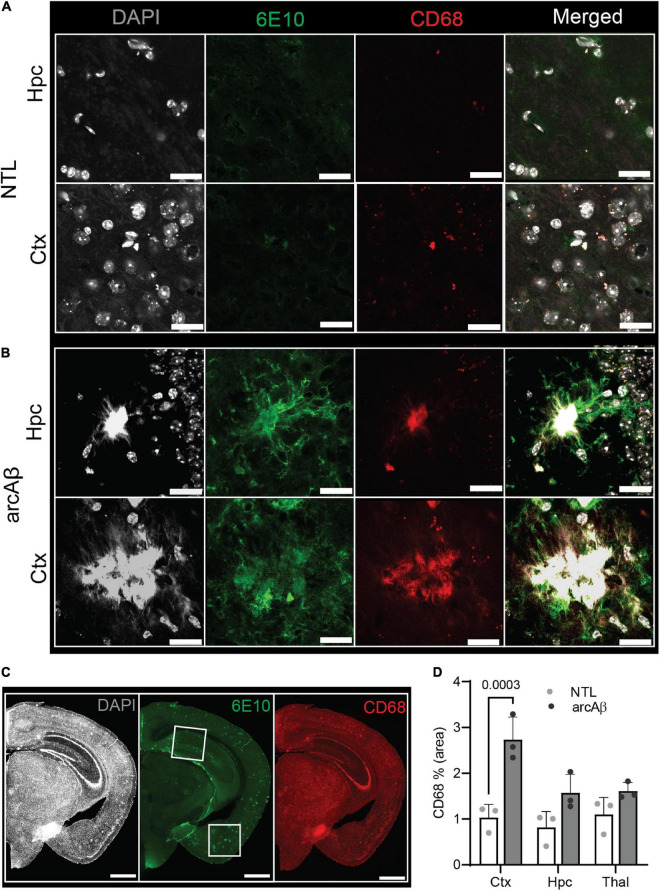
Microgliosis in the 17-month-old arcAβ mouse brain. **(A–C)** Brain tissue sections of non-transgenic littermate (NTL, *n* = 3) and arcAβ mice (*n* = 3) were stained for 6E10 (green)/CD68 (red) in the hippocampus (Hpc) and cortex (Ctx). White boxes in panel **(C)** indicate the regions including cortex (Ctx), cornu ammonus 1 (CA1) region of the hippocampus (Hpc) that zoomed-in in panels **(A,B)**. Nuclei were counterstained with DAPI (white). Scale bar = 20 μm **(A,B)**, =1 mm **(C)**. **(D)** Quantification of CD68 signals in the Hpc, Ctx, and thalamus (Thal) of arcAβ mice compared to NTL mice confirmed microgliosis in arcAβ mice.

Next, the levels of activated microglia using Iba1 and CD68 and astrocytes using GFAP were assessed in the brains of arcAβ mice and NTL mice at 17 months of age (*n* = 3 per group). Increased numbers of microglia (Iba1% area) were observed in the vicinity of Aβ plaques and were upregulated in the cortex (3.99 ± 0.04 vs. 0.76 ± 0.68, *p* < 0.0001), hippocampus (4.44 ± 0.35 vs. 0.24 ± 0.33, *p* < 0.0001), and thalamus (3.81 ± 0.21 vs. 1.37 ± 0.80, *p* = 0.0001) of arcAβ mice at 17 months of age compared to NTL mice (*n* = 3 per group). Similarly, an increased GFAP% area was associated with plaque in the cortex (5.29 ± 1.57 vs. 0.56 ± 0.33, *p* < 0.0001) and hippocampus (5.75 ± 0.72 vs. 2.61 ± 0.53, *p* = 0.0021) of arcAβ mice at 17 months of age compared to NTL mice (*n* = 3 per group), but not in the thalamus (1.29 ± 0.87 vs. 0.70 ± 0.44, *p* = 0.7933) (Figures 1, 2C,D). CD68 is a lysosomal protein expressed at high levels by activated microglia and at low levels by resting microglia in the CNS. Reactive microglia indicated by increased CD68 surrounding amyloid plaques (6E10) were observed and increased in the cortex (2.74 ± 0.50 vs. 1.03 ± 0.29, *p* = 0.0003) of arcAβ mice at 17 months of age compared to NTL mice (*n* = 3 per group) and were comparable in the hippocampus (1.57 ± 0.40 vs. 0.82 ± 0.35, *p* = 0.0734) and thalamus (1.61 ± 0.19 vs. 1.10 ± 0.37, *p* = 0.2874) (Figure 3).

### No difference in whole-brain levels of [^18^F]RoSMA-18-d6 and [^11^C]RSR-056 specific binding or *Cnr2* expression between arcAß and non-transgenic littermate mice of different ages

Autoradiography using [^18^F]RoSMA-18-d6 (0.5 nM), [^11^C]RSR-056 (2 nM), and [^11^C]RS-028 (3 nM) was performed on sagittal arcAß brain tissue at 17 months of age slides to assess the radioligand specificity. The concentrations of the radioligands used in autoradiography were determined based on previous publication (at half binding affinity of the ligand) [^11^C]RS-028 (Haider et al., 2018), [^11^C]RSR-056 (Slavik et al., 2015), and [^18^F]RoSMA-18-d6 (Haider et al., 2020)Ḟor the brain tissues, less than 50% of binding sites were blocked in the presence of CB2R antagonist GW405833 (10 μM). The percentage of specific binding is significantly lower than percentages previously reported for the spleen for these radioligands, likely due to the limited number of CB_2_R binding sites in the brain (Slavik et al., 2015; Haider et al., 2018, 2020). [^18^F]RoSMA-18-d6 (40.3 ± 9.2%) showed a higher percentage of specific binding than [^11^C]RSR-056 (32.0 ± 7.8%) and [^11^C]RS-028 (32.0 ± 12.8%, Supplementary Figure 1) on arcAß (*n* = 3) and NTL (*n* = 3) mouse brain tissue at 17 months of age 3.

**FIGURE 4:**
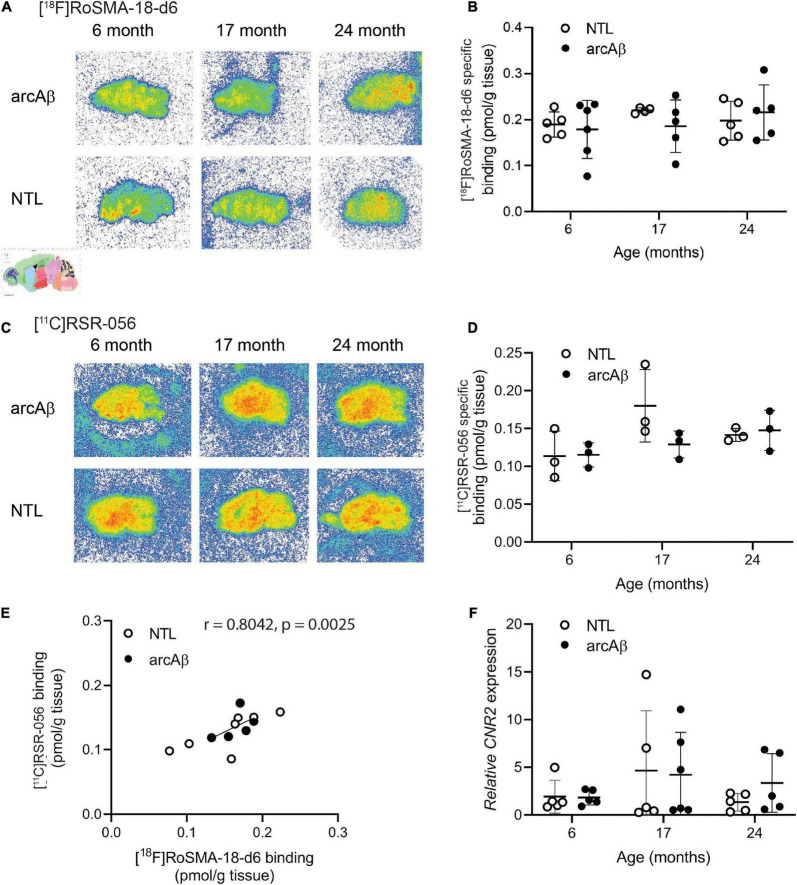
Comparable regional [^18^F]RoSMA-18-d6 and [^11^C]RSR-056 specific binding in the brains of arcAβ and non-transgenic mice at 6, 17, and 24 months of age. **(A)** Representative [^18^F]RoSMA-18-d6 (0.5 nM) autoradiographic images (total binding) of sagittal brain sections of arcAβ and non-transgenic littermate (NTL) mice at 6 months of age (*n* = 5, 6), 17 months of age (*n* = 4, 5), and 24 months of age (*n* = 5, 5). **(C)** Representative [^11^C]RSR-056 (2 nM) autoradiographic images (total binding) of sagittal brain sections of arcAβ and non-transgenic littermate (NTL) mice at 6 months of age (*n* = 3, 3), 17 months of age (*n* = 3, 3), and 24 months of age (*n* = 3, 3). **(B,D)** Quantification of specific binding of [^18^F]RoSMA-18-d6 (0.5 nM), and [^11^C]RSR-056 (2 nM) in the whole sagittal brain slice in panels **(A,C)**. Two-way ANOVA, arcAβ vs. NTL. **(E)** Robust correlation between specific binding of [^18^F]RoSMA-18-d6 (0.5 nM) and [^11^C]RSR-056 (2 nM) autoradiograms on arcAβ and NTL mouse brain hemispheres (Spearman rank, *r* = 0.8042, *p* = 0.0025). **(F)** *Cnr2* expression in arcAβ and NTL mouse brain hemisphere homogenates at 6 months of age (*n* = 5, 5), 17 months of age (*n* = 5, 6), and 24 months of age (*n* = 5, 5).

Thus, [^18^F]RoSMA-18-d6 (0.5 nM) and [^11^C]RSR-056 (2 nM) were selected for further experiments to examine the CB_2_R levels in arcAß and NTL at 6, 17, and 24 months of age by autoradiography of mouse brain slices. As no specific regional pattern of [^18^F]RoSMA-18-d6 (at 0.5 nM) and [^11^C]RSR-056 (at 2 nM) specific binding was observed in arcAß and NTL mice at 6, 17, and 24 months of age, the specific binding level was analyzed using the whole hemisphere region-of-interest. No difference was observed in brain [^18^F]RoSMA-18-d6 (at 0.5 nM) levels between NTL and arcAß mice at 6 months of age (0.19 ± 0.03 vs. 0.18 ± 0.06 pmol/g tissue, *n* = 5, 6), 17 months of age (0.22 ± 0.01 vs. 0.19 ± 0.01 pmol/g tissue, *n* = 3, 5), and 24 months of age (0.20 ± 0.04 vs. 0.22 ± 0.06 pmol/g tissue, *n* = 5, 5) (Figures 4A,B). Similarly, for [^11^C]RSR-056 (2 nM), no difference in radioactivity accumulation was observed in the brains of NTL and arcAß mice at 6 months of age (0.11 ± 0.03 vs. 0.12 ± 0.02 pmol/g tissue, *n* = 5, 6), 17 months of age (0.18 ± 0.05 vs. 0.13 ± 0.02 pmol/g, *n* = 3, 5), and 24 months of age (0.14 ± 0.09 vs. 0.15 ± 0.03 pmol/g, *n* = 5, 5) (Figures 4C,D). There was a robust correlation between [^11^C]RSR-056 (2 nM) specific binding and [^18^F]RoSMA-18-d6 (at 0.5 nM) specific binding in arcAß and NTL mouse brains at 6, 17, and 24 months of age (Spearman rank, *r* = 0.8042, *p* = 0.0025) (Figure 4E).

Next, the mRNA expression levels of *Cnr2* were evaluated in the left hemisphere from the same cohort of arcAß and NTL mice in autoradiography at 6, 17, and 24 months of age that were assessed by [^18^F]RoSMA-18-d6 and [^11^C]RSR-056 autoradiography (*n* = 5–6/age group). No significant difference was observed in *Cnr2* mRNA expression between the NTL and arcAß mice at 6 months of age (1.92 ± 1.72 vs. 1.83 ± 0.79), 17 months of age (4.65 ± 6.30 vs. 4.20 ± 4.43), and 24 months of age (1.33 ± 0.92 vs. 3.36 ± 3.07) (Figure 4F).

## Discussion

Here, we demonstrated an increase in local CB_2_R expression levels in arcAβ mice at 17 months of age compared to NTL mice and evaluated novel PET tracers [^11^C]RSR-056 and [^18^F]RoSMA-18-d6 for detecting brain CB_2_R changes in arcAβ mice at 6, 17, and 24 months of age. Increased CB_2_R fluorescence intensities and numbers of microglia and astrocytes inside/surrounding Aß plaques were observed in arcAß mice compared to NTL mice at 17 months of age. However, no significant difference in CB_2_R levels was observed at the whole-brain level measured either by using autoradiography or by mRNA analysis in arcAβ compared to NTL mice at 6, 17, and 24 months of age.

Cannabinoid type 2 receptor has been an emerging target for imaging neuroinflammation partly due to its low expression levels under physiological conditions and upregulation under acute inflammatory conditions (Stella, 2010). The CB_2_R fluorescence intensity was greatly increased in arcAβ mice compared to NTL mice and was higher inside plaque than peri-plaque and in the parenchyma of arcAβ mice. This observation is different from a previous publication of a significant increase in CB_2_R intensities compared to the core of plaques (radius ≤7 μm) (Savonenko et al., 2015). In addition, recent studies have reported the astroglial and neuronal expression of CB_2_R in mouse and rat brains in addition to its expression on microglia by using immunostaining and RNAscope techniques (Van Sickle et al., 2005; Gong et al., 2006; Zarruk et al., 2012; Li and Kim, 2015; Savonenko et al., 2015; Yamagishi et al., 2019; Galán-Ganga et al., 2021). Savonenko et al. (2015) reported expression of CB_2_R in neurons; and astrocytes in the brain from J20 amyloidosis mouse model at 12 months of age, in additional to its microglial expression. In our results from the immunofluorescence staining, CB_2_R expression on both astrocytes and microglia was increased significantly in arcAβ mice compared to the negligible level in NTL mice at 17 months of age (Figures 1, 2). Concerns have been raised regarding the specificity of CB_2_R antibodies used in immunohistochemical staining. Specific neuronal subpopulations of CB_2_R have been shown by using fluorescence *in situ* hybridization and proximity ligand assays in non-human primates (Sierra et al., 2015). However, previous study has reported that CB2-GFP expression is colocalized with Iba1 staining of microglia but not with NeuN staining of neuron or GFAP staining of astrocyte in CB2-GFP BAC transgenic mice (Lundt et al., 2015). López et al. (2018) reported that CB_2_R-dependent-enhanced green fluorescent protein (EGFP) expression is limited to plaque-associated microglial cells but is absent in neurons and astrocytes in CB2^EGFP/f/f/^5 × FAD mice.

Although Cnr2 expression in AD APP/PS1 has been reported to be upregulated, great variation between animals and a low fold increase lead to insignificance in comparison (Aso et al., 2013; Vidal-Palencia et al., 2022). Recent gene expression analysis showed that regional Cnr2 expression differs between male/female APP/PS1 mice (Vidal-Palencia et al., 2022). Here, Cnr2 expression was analyzed using homogenates of half hemispheres of arcAβ and NTL mice with further dissection. No difference in Cnr2 expression between arcAβ and NTL mice of different ages was observed.

For preclinical imaging, high variabilities in imaging of brain CB_2_R levels among animal models of neuroinflammation were reported from previous studies. Upregulated levels of brain CB_2_R have been demonstrated in transient middle cerebral artery occlusion ischemic stroke mice using [^18^F]RoSMA-18-d6 (Ni et al., 2021a) and in senescence-accelerated SAMP10 mice using [^11^C]NE40 (Yamagishi et al., 2019). Another study by PET using [^11^C]A-836339 in a lipopolysaccharide-injected rat model did not report changes in tracer uptake following neuroinflammation (Pottier et al., 2017). MicroPET using [^11^C] A-836339 showed increased uptake in the brain areas with Aβ depositions in a J20 mouse model of AD (Ni et al., 2019b). In the only reported study in patients with AD using PET, Ahmad et al. (2016) reported lower CB_2_R availability in Aβ-positive AD patients than in healthy controls assessed by PET using [^11^C]NE40 and [^11^C]PIB, respectively. No relationship between [^11^C]NE40 and cerebral Aβ load was observed in this study.

[^11^C]RSR-056 and [^18^F]RoSMA-18-d6 showed 32 and 40% specific binding in the AD mouse brain, respectively, and there was no difference between arcAβ and NTL mice. One of the difficulties is the low CB_2_R expression level in the brain and the low number of binding sites. Using the same tracers, [^11^C]RS-028 (Haider et al., 2018), [^11^C]RSR-056 (Slavik et al., 2015), and [^18^F]RoSMA-18-d6 (Haider et al., 2020), lower non-specific binding has been shown in post-mortem spleen and spinal cord tissues from patients with amyotrophic lateral sclerosis than in those from healthy controls. Further development of CB_2_R tracers of even higher affinity to overcome the low number of binding sites (Bmax) is desired. In addition, as species differences exist regarding CB_2_R brain expression, further studies on post-mortem brain tissues from patients with AD will provide information on CB_2_R disease relevance.

There are several limitations in this study: (1) Negative control: CB_2_R has been considered difficult to validate by using immunohistochemical staining. Further studies using CB_2_R knockout mice showing absence of staining and binding of radioligand will be confirmative. (2) Sample size: Number of animals included in the mRNA and autoradiography experiment of this brief report is low. Further study using larger sample size will improve the accuracy of the results. (3) *In vivo* imaging: Autoradiography provides information on the probe binding specificity and identifies potential regions of interest with validation from immunohistochemical characterization. Due to a lack of difference from autoradiography, *in vivo* measurement has not been performed in the AD mouse models using the radioligands investigated.

## Conclusion

In conclusion, increases in CB_2_R immunofluorescence intensity on the glia were detected in the brains of arcAβ mice compared to NTL mice and were associated with Aβ deposits. Further improvement of the binding properties of CB_2_R PET tracers will be needed to detect subtle changes in CB_2_R in an AD animal model.

## Data availability statement

The datasets presented in this study can be found in online repositories. The names of the repository/repositories and accession number(s) can be found in the article/Supplementary material.

## Ethics statement

The animal study was reviewed and approved by the Cantonal Veterinary Office Zurich.

## Author contributions

RN, JK, SA, and AHa designed the study. VK performed the staining and microscopy. LM synthesized the radioligands. FS performed the mRNA analysis. RN performed the autoradiography. VK, FS, LM, and RN performed data analysis. VK and RN wrote the initial manuscript. All authors read and approved the final manuscript.
